# Long-term health-related quality of life and burden of disease after intensive care: development of a patient-reported outcome measure

**DOI:** 10.1186/s13054-021-03496-7

**Published:** 2021-02-25

**Authors:** Johan Malmgren, Ann-Charlotte Waldenström, Christian Rylander, Elias Johannesson, Stefan Lundin

**Affiliations:** 1 Department of Anaesthesiology and Intensive Care Medicine, Institute of Clinical Sciences, Sahlgrenska Academy, Sahlgrenska University Hospital, University of Gothenburg, Blå Stråket 5, 413 45 Gothenburg, Sweden; 2 Department of Oncology, Institute of Clinical Sciences, Sahlgrenska Academy, Sahlgrenska University Hospital, University of Gothenburg, 413 45 Gothenburg, Sweden; 3 Department of Social and Behavioural Studies, University West, Trollhättan, Sweden

**Keywords:** Critical care, Intensive care unit, Critical illness, Quality of life, Follow-up studies, Long-term adverse effects, Questionnaire, Patient-reported outcome, Survivors, Survivorship

## Abstract

**Background:**

ICU survivorship includes a diverse burden of disease. Current questionnaires used for collecting information about health-related problems and their relation to quality of life lack detailed questions in several areas relevant to ICU survivors. Our aim was to construct a provisional questionnaire on health-related issues based on interviews with ICU survivors and to test if this questionnaire was able to show differences between ICU survivors and a control group.

**Methods:**

Thirty-two ICU survivors were identified at a post-ICU clinic and interviewed at least six months after ICU discharge. Using an established qualitative methodology from oncology, all dysfunctions and disabilities were extracted, rephrased as questions and compiled into a provisional questionnaire. In a second part, this questionnaire was tested on ICU survivors and controls. Inclusion criteria for the ICU survivors were ICU stay at least 72 h with ICU discharge six months to three years prior to the study. A non-ICU-treated control group was obtained from the Swedish Population Register, matched for age and sex. Eligible participants received an invitation letter and were contacted by phone. If willing to participate, they were sent the questionnaire. Descriptive statistics were applied.

**Results:**

Analysis of the interviews yielded 238 questions in 13 domains: cognition, fatigue, physical health, pain, psychological health, activities of daily living, sleep, appetite and alcohol, sexual health, sensory functions, gastrointestinal functions, urinary functions and work life. In the second part, 395 of 518 ICU survivors and 197 of 231 controls returned a completed questionnaire, the response rates being 76.2% and 85.3%, respectively. The two groups differed significantly in 13 of 22 comorbidities. ICU survivors differed in a majority of questions (p ≤ 0.05) distributed over all 13 domains compared with controls.

**Conclusions:**

This study describes the development of a provisional questionnaire to identify health-related quality of life issues and long-term burden of disease after intensive care. The questionnaire was answered by 395 ICU survivors. The questionnaire could identify that they experience severe difficulties in a wide range of domains compared with a control group.

*Trial registry* ClinicalTrials.gov Ref# NCT 02767180

## Introduction

ICU survivorship may come at a price—the price of cognitive [1] and physical dysfunction [2], psychiatric and psychological problems [3], financial and work-related shortcomings [4] and healthcare consumption [5].

To identify and describe these problems, the intensive care community uses a synthesis of tests, examinations and questionnaires depending on the context: SF-36 and EQ-5D are the most commonly used questionnaires for measuring health-related quality of life [6, 7], but concerns regarding their ability to identify issues valued by ICU survivors have been raised [8]. Within the domains of physical, cognitive and mental health, the concept of PICS (post-intensive care syndrome) points out directions for investigations rather than provides scales. All three of these domains have numerous specific measurements, with for example at least 26 different tools to measure functional outcome [9]. Furthermore, discriminating post-ICU issues from prevalent psychological and physical ill-being in the general population is challenging when problems overlap [10, 11].

We hypothesized that a questionnaire mainly based on interviews with ICU survivors would contain a majority of issues experienced after intensive care, as well as carry a discriminative capacity to identify those issues with a magnitude distinct from a non-ICU-treated population. Influenced by advances made in oncology [12], we applied an established qualitative methodology [13, 14], using interviews to identify and extract issues from survivors. In an attempt to encase the full extent of the problems, interviews were not limited to particular areas.

Our aim was to develop a provisional questionnaire based on the content of such interviews, test its practicality in a scientific setting, as well as its ability to identify differences in the magnitude of issues between ICU survivors and non-ICU-treated controls. This would be a first step toward a questionnaire for long-term follow-up after intensive care. It could be useful to healthcare providers in post-ICU clinics as well as in primary care to identify clinical problems in need of specific treatments, consulting or referral to rehabilitation. In a research setting, it could be practical as an outcome measurement when evaluating issues and their trajectories.

Comparing the results from an ICU survivor group with those from a non-ICU-treated group gives two advantages. First, a comparison between an ICU survivor group and a non-ICU-treated group will aid a future reduction in the number of items. Second, by being able to measure the degree to which issues are related to intensive care and not to problems common in a non-ICU-treated population, the questionnaire could be used to identify domains suitable for interventional trials.

## Methods

### Interviews and development of a provisional questionnaire

#### Methodological framework and considerations

We took a pragmatic approach inspired by our earlier experience of developing instruments in oncology based on interviews [13, 14]. Thus, the method applied in this study follows recommendations of EORTC (European Organization of Research and Treatment of Cancer) and the Division of Clinical Cancer Epidemiology, Gothenburg, Sweden [15, 16].

While the methodology has similarities with Grounded Theory such as using data saturation as an endpoint and the parallel process of data collection and analyses, there are important differences. For example, we use an interviewer with clinical experience and domain knowledge. As recommended by the EORTC findings from the literature, other scales and questionnaires may be shown to the interviewee to evoke further thoughts in the second part of the interview. In summary, our methodology aims at creating an as comprehensive list of symptoms and issues as possible, where keeping the exact wording of the interviewees is important to minimize interpretations.

#### Setting and study population

Our post-ICU (16-bed mixed ICU in a university hospital) plans a scheduled visit six months after ICU discharge for survivors with an ICU length of stay of at least 72 h. Survivors may also contact the clinic for a visit or be invited at the discretion of the post-ICU clinic nurses. All survivors visiting the post-ICU clinic between February and May in 2015, with at least six months from ICU discharge, were eligible for the study.

#### Sampling strategies

Using a purposive, maximum variation sampling approach, potentially “information-rich” interviewees representative for different ages, gender, admission diagnoses, ICU length of stay, time from ICU discharge and postal areas as a marker of socioeconomic status were invited to participate [17]. They were selected by one of the researchers (J.M.), who had not been involved in their care but met them at their visit to the post-ICU clinic to gain trust and explain the study. Sample size was based on data saturation—the point where no new information emerged [18]. To confirm saturation, we decided a priori to continue the data collection for an additional three interviews.

#### Interviews

Interviews were conducted either in the post-ICU clinic or in their home, based on the interviewee’s own choice. There was no time limitation for the interviews, and participants were interviewed only once. Using a semi-structured technique, we explored their current situation as well as symptoms, difficulties, quality-of-life issues and social effects arising at any point after ICU discharge. All interviews started with the question “*We are asking for your help in creating a questionnaire which will be used to identify and follow the experiences of patients who have survived intensive care. I would like to ask you a few things about your health. Can you tell me about the experiences you may have had as a result of your intensive care stay, and the time between discharge and today?*”. While initial questions were open-ended, as interviews progressed details about findings were sought for. Once the interviewee could think of nothing further, domains and issues from previous interviews, literature or other scales and questionnaires were discussed (Additional file 1: Table S1). Examples of interview questions, probes and prompts can be seen in Additional file 4: Figure S1. Field notes were taken by the interviewer and read to the interviewee at the end of the interview to allow for comments or corrections.

#### Data analysis

In parallel with conducting the interviews, already transcribed interviews were independently analyzed by two of the researchers (J.M. and A-C.W.). Analyses were made manually: Long quotes were shortened while preserving the core meaning of the issues [16]. All extracted issues were categorized into domains, and duplicates were removed. To ensure that important issues were retained as well as to minimize the risk of recall bias, issues only had to be mentioned once to be included in the provisional questionnaire. No items were excluded in this phase since an item reduction will be performed at a later stage. The remaining issues were rephrased as questions, where care was taken to maintain the wording used by the interviewee.

At the time of data analysis, the first researcher (J.M.), a male intensive care physician, had two years of experience in qualitative research and several years of experience with post-ICU care. The second researcher (A-C.W.), a female gynaecological oncologist, had ten years of experience in qualitative research in the Division of Clinical Cancer Epidemiology, Gothenburg University, Sweden and 14 years of experience in the EORTC Quality of Life Group.

#### Additional questions

Composite questions about domain-specific quality of life and domain-specific future concerns were added at the end of each domain (*How much do you think problems within [domain] affects your quality of life? For the past month, have you been worried about your future regarding [domain]?)*. Empty space and a request for missing issues or other comments were provided after each domain.

Questions regarding demographics and comorbidities were added at the end of the questionnaire.

#### Response scales

The response scales used are based on the established experience of the Division of Clinical Cancer Epidemiology, Gothenburg, Sweden, and were created to match each conceptual entity as closely as possible using incidence, prevalence, intensity and agreement when applicable (Table 1) [13].

**Table 1:** Examples of wordings and response scales of questions asked in the questionnaire

Measure	Question	Response scale
Incidence	Have you had leakage of stools because of not being able to reach the toilet in time?	No—Occasionally—Once a week—Several times a week—Once a day—Several times a day
Prevalence	Have you needed help moving between chair and bed	No—Occasionally—Half of the times—Most of the times—Every time
Intensity	Have you found normal touch bothersome?	Not at all—A little—Moderately—Quite a bit—Very much
Agreement	Have you had difficulties extending your wrist?	No—Yes

Because our interest lies in long-term effects, the time frame asked about in most response scales was "the last month." This would also minimize the problem of recall bias. Care was taken not to overlap between alternatives and to include "Not applicable" if needed.

#### Content validity and cognitive interviews

Evidence of content validity as a measurement property refers to the extent of which an instrument contains the relevant aspects of the construct it intends to measure, in our case “issues experienced after intensive care” [19]. However, the content is not only the issues raised within the questions, but also such aspects as the wording of questions, the clarity of instructions and proper response scales [18]. In accordance with the ISPOR (International Society for Pharmacoeconomics and Outcomes Research) guidelines on testing for evidence of content validity, all questions were tested with cognitive interviews on additional ICU survivors chosen with the same criteria as the initial interviewees, and with the same saturation-based sample size [20]. These interviews were recorded as well. The cognitive interviews were the final opportunity to make content changes before administering the questionnaire to a larger group and included appropriate response scales and recall period. The aim was to ensure that the questions were conceptually clear, easily understood, perceived as relevant and to make sure no important issues are missing. Interviewees were initially instructed to complete the questionnaire while thinking aloud, but as the two first interviewees failed to follow these instructions, we changed to a retrospective probing technique, where questions were asked after finishing each domain, in line with EORTC’s guidelines when a questionnaire has a substantial number of questions [15].

### Application of the questionnaire

Eligible patients were all adult ICU survivors admitted between February 2013 and December 2015 to one of three mixed ICUs in Sahlgrenska University Hospital, Gothenburg, Sweden (in total 31 ICU beds), and with a minimum ICU length of stay of 72 h. They all had been discharged from the ICU between six months and three years prior to the study. Exclusion criteria were primary neurological/neurosurgical reason for admission, limited understanding of Swedish as judged by study personnel, no Swedish personal identity number, no Swedish address or phone number or a secret Swedish personal identity. We obtained a non-ICU-treated control group from the Swedish Population Register, matched for age and sex with respect to ICU survivors having returned a completed questionnaire. For the version of the questionnaire addressing the control group, we removed all questions requiring a previous ICU stay (e.g., *Have you had difficulties describing your ICU experiences?*) and added one question checking for previous intensive care. Exclusion criteria for the control group were previous ICU stay or a limited understanding of Swedish.

All eligible participants received an initial letter with information about the study, and within a week they received a phone call asking for participation. The questionnaire was sent together with a pre-paid return envelope, and reminder phone calls were made if the questionnaire was not returned within two weeks. The questionnaire was sent to the ICU survivors between April 2016 and October 2017, and to the control group between March 2017 and December 2017.

### Statistical analysis

Univariate descriptive statistics are presented as frequencies and percentages for all categorical variables. Continuous variables were screened for normality using Shapiro-Wilks (p > 0.05) and box-plots. For non-normally distributed continuous variables, median and range or median and interquartile range (IQR) are reported. Bivariable comparisons were made between ICU survivors and the control group for all ordered categorical variables and continuous variables in order to identify differences between the groups by applying the Mann–Whitney U test. These results are presented as means and mean rank sums and the associated p-value calculated in the Mann–Whitney U test. In addition, all the bivariable comparisons for ordered categorical variables were analyzed with Fisher's exact test as a robustness check. Dichotomous variables were also assessed with Fisher's exact test. All tests were two-tailed, and significance level was set to 0.05.

Questionnaires were scanned with Remark Office OMR (Remark Office OMR 10, Gravic Inc, Malvern, USA). Statistical analyses were performed using the IBM SPSS v26 package (IBM SPSS v26 Statistics, IBM, Armonk, USA).

## Results

### Interviews and development of a provisional questionnaire

#### Study population

The median age of the interviewees was 55.5 years (range 20–82), and 33% were females. The interviews took place at a median of 14.7 months (range 7.6–68.0) after ICU discharge. The median ICU length of stay was 4.9 days (range 1.7–76.1), and the median SAPS 3 score was 57.5 (range 24–81). Seventy per cent were treated with mechanical ventilation for a median time of five days (range 1–62). The most common primary diagnosis was infection/sepsis (18.8%), followed by trauma and cardiac arrest as second and third most common (both 12.5%) diagnosis (Table 2).

**Table 2:** Clinical characteristics of ICU survivors and interviewees

	ICU survivors (n = 395)	Interviewees (n = 32)
*Primary diagnosis, n (%)*		
Infection/sepsis	110 (27.8)	6 (18.8)
Trauma	53 (13.4)	4 (12.5)
Respiratory failure	43 (10.9)	3 (9.4)
Major bleeding^1^	38 (9.6)	0 (0.0)
Cardiac arrest	24 (6.1)	4 (12.5)
GI diseases^2^	17 (4.3)	3 (9.4)
Liver failure	16 (4.1)	1 (3.1)
Transplantation	16 (4.1)	3 (9.4)
Postoperative	16 (4.1)	1 (3.1)
Renal failure	12 (3.0)	3 (9.4)
Pulmonary diseases^3^	10 (2.5)	0 (0.0)
Cardiac failure and/or myocardial infarction	10 (2.5)	2 (6.3)
Vascular disorders^4^	10 (2.5)	0 (0.0)
Metabolic disorders	8 (2.0)	0 (0.0)
Other	7 (1.8)	2 (6.3)
Oncological or haematological disorders	5 (1.3)	0 (0.0)
*SAPS3 score*		
Median (range)	59 (16–100)	57.5 (24–81)
*ICU, length of stay*		
Days, median (range)	5.5 (3.0–78.6)	4.9 (1.7–76.1)
*Mechanical ventilation*		
Patients, *n* (%)	310 (78.5)	23 (69.7)
Days, median (range)	5.0 (1.0–74.0)	5 (1.0–62.0)
*Continuous renal replacement therapy*		
Patients, *n* (%)	88 (22.3)	11 (33.0)
Days, median (range)	6.0 (1.0–42.0)	8.0 (3.0–53.0)

^1^including aortic rupture and GI bleeding; ^2^including pancreatitis and peritonitis; ^3^including COPD and pneumonitis; ^4^ including emboli and thrombi

#### Interviews

All invited patients accepted to be interviewed. In total, 32 interviews including six cognitive interviews were performed. Ten of the interviews were conducted in the presence of a partner. Apart from one interview conducted in the interviewee’s home and one conducted in a public location, all interviews were conducted in the post-ICU clinic. The average time of interviews was 49 min (range 15–113). Minor language corrections were made based on the cognitive interviews, but no new issues were identified. No question was considered upsetting, no response scale was changed, and no time frame was adjusted.

#### Data analysis

Quotes from the interviews generated 437 issues. By removing duplicates and similarities, they were reduced to 195 unique issues (Additional file 5: Figure S2). These were rephrased as questions and categorized into 13 domains: cognition, fatigue, physical health, pain, psychological health, activities of daily living, sleep, appetite and alcohol, sexual health, sensory functions, gastrointestinal functions, urinary functions and work life.

#### Additional questions

For the questionnaire, 31 composite questions regarding domain-specific quality of life and domain-specific future worries were added. Twelve questions from other scales and questionnaires were considered relevant by the interviewees and were added: All three questions from AUDIT-C [21], four questions from the KATZ-ADL index [22], four questions from the Work Ability Index [23] and one question about the ability to walk for six minutes [24]. The distribution of questions is shown in Table 3**.** In the version of the questionnaire for the control group, twenty questions requiring a previous ICU-stay were removed.

**Table 3:** Domains and number of questions in the questionnaire sent to ICU survivors

Domain	Number of questions
Cognitive function	31
Fatigue	14
Physical health	31
Pain	19
Psychological aspects	29
Activities of daily living (ADL)	16
Sleep	11
Appetite and alcohol	11
Sexual health	14
Sensory functions	26
Gastrointestinal functions	7
Urinary functions	8
Work life	21
Total number of questions	238

#### Response scales

A majority of questions was measured on an ordered category scale: 113 questions on a 6-point scale, 91 questions on a 5-point scale, eight questions on a 4-point scale, two questions on a 3-point scale. Twenty-two questions were measured on a dichotomous scale and two questions were quantitative. Higher scores indicated higher levels of difficulties or problems except in eleven reversely coded questions where higher scores indicated lower levels of problems (e.g., *Do you have the ability to look forward to things?*; *No—Rarely—Sometimes—Quite often—Very often—All the time*).

### Application of the questionnaires

**Fig. 1:**
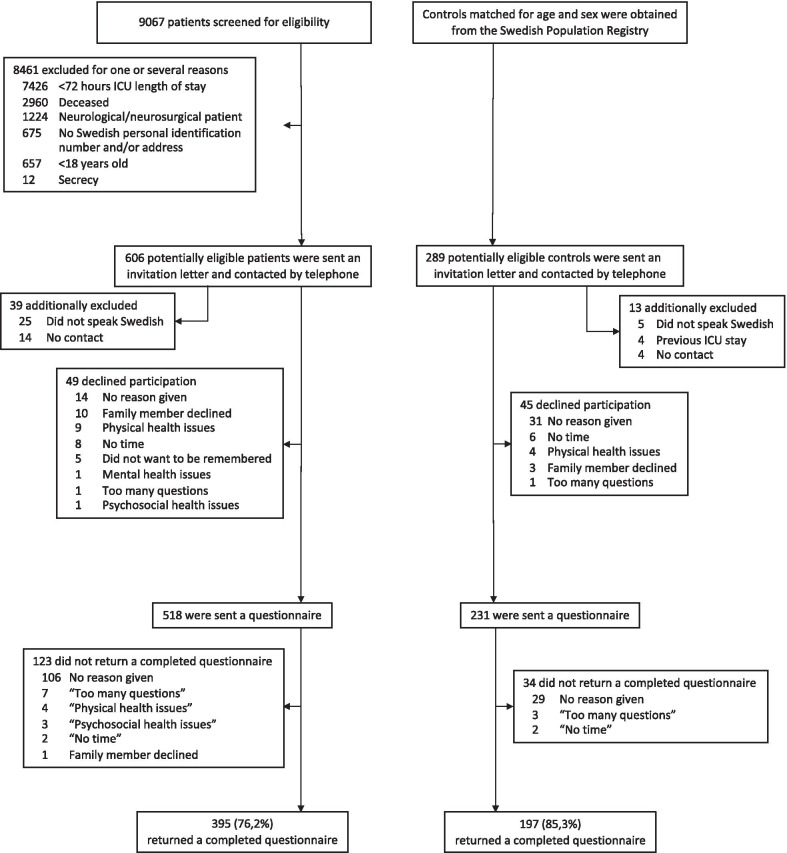
CONSORT diagram of screening, recruitment, follow-up and reasons for non-participation

A total of 518 ICU survivors and 289 controls received a questionnaire. Among these, 395 ICU survivors and 195 controls returned a completed questionnaire, the return rates being 76.2% and 85.3%, respectively (Fig. 1). The most commonly stated reason for declining participation among ICU survivors was family members declining (1.8%; 10 of 567) and among controls the reason was "No time" (2.2%; 6 of 276).

The most frequent ICU admission diagnosis for survivors was infection/sepsis (27.8%; n = 110) followed by trauma (13.4%; n = 53) and respiratory failure (10.9%; n = 43). Median SAPS 3 score was 59 (range 16–100), and median ICU length of stay was 5.6 days (range 3.0–78.6). Most ICU survivors were mechanically ventilated (78.5%), with a median time of 4.0 days (range 0–74). The representation of the major diagnosis groups was fairly similar between the ICU survivors and the interviewees (Table 3).

#### Demographics and comorbidities

There were no differences in age and gender between the ICU survivors and the control group (Table 4). While there were no differences in educational levels between the two groups, significantly more ICU survivors were on sick leave/sickness benefit compared to the control group (p = 0.000). The ICU survivors were also sicker compared with controls, differing significantly in 13 of 22 comorbidities (Table 5). Cardiovascular disease (hypertension, angina pectoris, myocardial infarction and heart failure) was more common among the ICU-survivors as was respiratory disease and pulmonary embolus. The ICU survivors suffered more often from depression and anxiety. Diabetes, kidney disease and bowel disease were also more common in this group. The need for walking aids or wheelchair due to physical impairment occurred only among ICU survivors as did having amputated limb(s).

**Table 4:** Demographics for ICU survivors and controls at the time of answering the questionnaire

	ICU survivors (n = 395)	Controls (n = 195)	p-value	Total (N)
Age, years; median (IQR)	65.0 (18)	65.0 (15)	0.56	589
Body mass index; median (IQR)	26.0 (7)	25.4 (5)	0.17	555
Smoker; n (%*)	15 (13)	15 (11)	0.01	109
Male; n (%*)	239 (61)	117 (60)		
Education; n (%*)			0.132	574
Primary school	161 (42)	64 (33)		
Secondary school	85 (22)	47 (24)		
College or University	125 (33)	73 (38)		
Other	10 (3)	9 (5)		
Employment status; n (%*)			0.000	570
Contract and self-employment, parental leave	79 (21)	86 (45)		
Sickness benefit/sick leave	88 (23)	5 (3)		
Unemployed (out of work)	2 (1)	1 (1)		
Student	2 (1)	0 (0)		
Retired	206 (55)	101 (52)		
Current form of living; n (%*)			0.00	575
Hospital	2 (1)	0 (0)		
Rehab	3 (1)	0 (0)		
Nursing home	3 (1)	0 (0)		
Residential home	13 (3)	1 (1)		
Apartment	187 (49)	53 (28)		
Detached house	176 (46)	137 (72)		
Civic status, n (%*)				
Married/partner	233 (63)	159 (84)	0.00	545

* percent of responding participants

**Table 5:** History of comorbidities for ICU survivors and controls at the time of answering the questionnaire

	ICU survivors (n = 395)	Controls (n = 195)	p-value
Cardiovascular, n (%*)			
Hypertension	183 (50)	60 (34)	0.00
Angina pectoris	28 (8)	5 (3)	0.03
Myocardial infarction	52 (15)	4 (2)	0.00
Heart failure	56 (16)	11 (6)	0.00
Respiratory, n (%*)			
Lung disease, e.g., COPD, bronchitis	50 (14)	5 (3)	0.00
Pulmonary embolus	21 (6)	1 (1)	0.00
Asthma	34 (9)	19 (11)	0.54
Sleep apnoea	35 (10)	15 (8)	0.64
Home ventilator	7 (2)	0 (0)	0.10
Neurological, n (%*)			
Stroke	34 (9)	11 (6)	0.25
Dementia/Alzheimer's disease	2 (1)	3 (2)	0.36
Multiple sclerosis	2 (1)	2 (1)	0.60
Parkinson's disease	3 (1)	1 (1)	1.00
Psychiatric, n (%*)			
Psychological diseases, e.g., depression, anxiety	65 (19)	17 (8)	0.01
Metabolic, n (%*)			
Non-insulin-dependent diabetes	53 (15)	14 (8)	0.03
Insulin-dependent diabetes	43 (11)	4 (2)	0.00
Kidney disease	33 (8)	3 (2)	0.00
Dialysis	6 (2)	1 (1)	1,00
Other, n (%*)			
Tumour disease	44 (13)	14 (8)	0.14
Bowel disease	42 (12)	10 (6)	0.03
Rheumatic disease	27 (8)	9 (5)	0.28
Physical walking aids, n (%*)			
Walking stick/crutches	0 (0)	0 (0)	N/A
Walking frame/rollator	69 (18)	0 (0)	0.00
Wheelchair/electric wheelchair	35 (9)	0 (0)	0.00
Bedridden	5 (1)	0 (0)	0.18
Amputated limb(s)	13 (3)	0 (0)	0.01

*Percent of responding participants

#### Symptoms and burden of disease

At the time of completing the questionnaire, which was between six months and three years after discharge, the ICU survivors differed significantly in a majority of questions across all domains when compared with the control group (Additional file 2: Table S2a and Table 6, 7, 8, 9, 10, 11, 12, 13, 14, 15, 16, 17, 18). Examples are:

**Table 6:** Means and mean rank sums for the domain on cognition—Comparison between ICU survivors and controls at the time of completing the questionnaire

	Issues	Mean		Mean rank sum		*p*-value
		ICU survivors	Controls	ICU survivors	Controls	
Cog1	Difficulties finding words	2.10	1.98	283.92	278.15	0.677
Cog2	Difficulties finishing sentences	1.72	1.47	291.65	265.66	0.040
Cog3	Losing the thread easily	1.88	1.46	302.73	246.23	0.000
Cog4	Don't remember what you have said	1.68	1.44	293.57	260.18	0.008
Cog5	Don't remember what you have done	1.46	1.22	294.28	257.11	0.001
Cog6	Think you have done something but you haven't	1.44	1.20	296.86	256.42	0.000
Cog7	Forgotten what you were going to get	2.30	2.11	283.64	272.82	0.427
Cog8	Need to be reminded to do an activity	1.57	1.29	265.27	236.39	0.007
Cog9	Difficulties thinking clearly	1.93	1.60	295.96	259.70	0.007
Cog10	Need for memos	2.40	2.27	284.43	275.63	0.529
Cog11	Difficulties remembering names	2.39	2.36	283.09	285.84	0.845
Cog12	Difficulties remembering general knowledge	1.69	1.63	253.68	247.39	0.599
Cog13	Difficulties remembering what you have read	1.95	1.77	272.88	260.12	0.324
Cog14	Difficulties remembering previous TV-episode	1.67	1.45	230.68	211.66	0.078
Cog15	Difficulties learning new things	1.71	1.50	230.61	219.21	0.287
Cog16	Difficulties remembering numbers	1.76	1.70	269.08	268.84	0.984
Cog17	Difficulties being on time	1.28	1.17	270.79	258.38	0.127
Cog18	Missed a scheduled meeting	1.14	1.04	260.82	249.87	0.064
Cog19	Mistaken which day of the week	1.77	1.41	297.31	251.46	0.000
Cog20	Forgotten where you have put something	2.26	2.34	273.42	297.58	0.082
Cog21	Need to double-check things	2.09	2.08	277.60	287.75	0.461
Cog22	Difficulties finding your way around	1.51	1.38	286.37	274.76	0.263
Cog23	Someone has said that you have memory problems	1.59	1.29	295.43	259.51	0.002
Cog24	Worrying about having memory problems	1.61	1.43	288.59	270.33	0.118
Cog25	Difficulties taking initiatives	1.89	1.53	298.98	252.38	0.000
Cog26	Difficulties prioritizing	1.73	1.59	245.72	235.74	0.378
Cog27	Difficulties concentrating	2.04	1.72	293.39	261.40	0.017
Cog28	Difficulties finding alternative solutions	1.59	1.34	234.77	208.96	0.010
Cog29	Time spent reading*	2.81	2.91	274.23	288.36	0.311
Cog30	Memory/thinking difficulties affecting QoL	1.80	1.40	262.02	214.63	0.000
Cog31	Worrying about your memory/thinking	1.78	1.52	295.25	266.15	0.021

*Reversely coded response scale

**Table 7:** Means and mean rank sums for the domain on fatigue—Comparison between ICU survivors and controls at the time of completing the questionnaire

	Issues	Mean		Mean rank sum		*p*-value
		ICU survivors	Controls	ICU survivors	Controls	
Fat1	Need for daytime rest	2.99	2.08	316.07	216.41	0.000
Fat2	Tough getting started doing things	2.72	2.09	308.67	234.46	0.000
Fat3	Difficulties finishing things due to feeling exhausted	2.07	1.49	306.05	234.26	0.000
Fat4	Difficulties doing things under pressure of time	2.27	1.57	219.22	170.91	0.000
Fat5	Difficulties multitasking due to feeling exhausted	2.00	1.43	304.51	234.61	0.000
Fat6	Tired from reading	1.86	1.41	274.08	228.4	0.000
Fat7	Tired from watching TV	1.79	1.48	289.96	251.01	0.002
Fat8	Tired from conversation between more than two people	1.96	1.54	286.09	238.79	0.000
Fat9	Fallen asleep when reading	1.51	1.48	253.62	257.66	0.714
Fat10	Fallen asleep during a conversation	1.08	1.09	284.11	283.77	0.956
Fat11	Tiredness affecting work	2.00	1.34	113.48	82.88	0.000
Fat12	Tiredness limiting social activities	2.06	1.55	224.71	171.19	0.000
Fat13	Tiredness affecting QoL	2.41	1.66	223.54	152.44	0.000
Fat14	Worrying about feeling tired	2.04	1.41	313.89	239.87	0.000

**Table 8:** Means and mean rank sums for the domain on physical health—Comparison between ICU survivors and controls at the time of completing the questionnaire

	Issues	Mean		Mean rank sum		*p*-value
		ICU survivors	Controls	ICU survivors	Controls	
Phys1	Physical health in general	3.29	2.63	319.92	214.59	0.000
Phys2	Reduced feeling in your face	1.16	1.04	289.48	276.21	0.028
Phys3	Arm weakness	2.00	1.36	315.44	223.91	0.000
Phys4	Reduced feeling in arms	1.42	1.10	303.37	253.11	0.000
Phys5	Reduced feeling in hands/fingers	1.61	1.19	307.10	242.97	0.000
Phys6	Raynaud's in fingers	1.92	1.51	296.24	262.93	0.008
Phys7	Difficulties lifting/carrying lightweight objects	1.52	1.13	302.42	249.40	0.000
Phys8	Difficulties turning on taps/opening jars	1.66	1.25	303.28	244.97	0.000
Phys9	Difficulties using your hands	1.67	1.24	311.16	245.96	0.000
Phys10	Leg weakness	2.31	1.40	329.21	205.79	0.000
Phys11	Reduced feeling in legs	1.74	1.11	314.39	231.29	0.000
Phys12	Reduced feeling in feet/toes	1.88	1.18	315.03	228.25	0.000
Phys13	Restless legs	1.75	1.31	303.90	248.40	0.000
Phys14	Dizziness when standing up	2.10	1.57	307.51	241.49	0.000
Phys15	Losing balance easily	2.17	1.39	308.52	215.49	0.000
Phys16	Difficulties climbing stairs	2.53	1.32	314.03	200.02	0.000
Phys17	Unsteady gait	1.88	1.23	302.06	229.12	0.000
Phys18	Legs feeling heavy	1.85	1.19	311.15	231.48	0.000
Phys19	Swollen legs/ankles	2.02	1.36	309.22	243.61	0.000
Phys20	Raynaud's in toes	2.02	1.24	311.81	234.25	0.000
Phys21	Contractures	1.98	1.46	305.16	239.26	0.000
Phys22	Periods of heavy sweating	1.66	1.40	295.38	264.13	0.006
Phys23	Shortness of breath limiting your physical activities	2.29	1.34	280.41	191.11	0.000
Phys24	Physically active > 30 min*	3.03	3.82	258.46	342.76	0.000
Phys25	Physical health affecting QoL	2.82	1.70	295.22	172.35	0.000
Phys26	Worrying about physical health	2.75	1.86	318.12	224.90	0.000

*Reversely coded response scale

**Table 9:** Means and mean rank sums for the domain on pain—Comparison between ICU survivors and controls at the time of completing the questionnaire

	Issues	Mean		Mean rank sum		*p*-value
		ICU Survivors	Controls	ICU Survivors	Controls	
Pain1	Headaches	1.85	1.73	288.44	281.10	0.585
Pain2	Finding normal touch bothersome	1.32	1.11	295.22	263.18	0.000
Pain3	General body pain	2.15	1.72	297.42	252.66	0.001
Pain4	Shoulder pain	2.29	1.92	296.63	263.25	0.014
Pain5	Arm pain	1.78	1.52	291.89	271.15	0.083
Pain6	Hand pain	1.67	1.40	294.48	267.39	0.017
Pain7	Back pain	2.57	2.16	295.79	264.91	0.027
Pain8	Chest pain	1.56	1.23	300.36	254.37	0.000
Pain9	Abdominal pain	1.74	1.36	299.65	254.35	0.000
Pain10	Leg pain	2.27	1.68	303.53	248.04	0.000
Pain11	Foot pain	2.09	1.48	304.52	246.06	0.000
Pain12	Pain stopping planned activity	2.12	1.46	309.61	240.04	0.000
Pain13	Painkillers to manage ADL	2.11	1.57	298.77	260.60	0.002
Pain14	Painkillers for sufficient sleep	1.88	1.32	302.72	251.32	0.000
Pain15	Pain makes going to sleep difficult	2.03	1.45	302.65	249.80	0.000
Pain16	Woken by pain	1.90	1.51	297.88	259.31	0.002
Pain17	Pain affecting QoL	2.74	1.98	257.81	182.52	0.000
Pain18	Worrying about pain	2.30	1.66	305.36	246.40	0.000

**Table 10:** Means and mean rank sums for the domain on psychological health—Comparison between ICU survivors and controls at the time of completing the questionnaire

	Issues	Mean		Mean rank sum		*p*-value
		ICU Survivors	Controls	ICU Survivors	Controls	
Psych1	Crying easily	1.83	1.39	303.02	256.37	0.000
Psych2	Feeling short-tempered	2.14	1.70	301.10	257.60	0.001
Psych3	Loosing patience easily	2.04	1.65	300.93	260.57	0.003
Psych4	Difficulties feeling warmth toward family members	1.59	1.39	290.76	278.08	0.255
Psych5	Difficulties unwinding	2.03	1.73	296.92	268.76	0.034
Psych6	Worrying about little things	2.11	1.75	298.82	261.93	0.007
Psych7	Feeling low-spirited	2.63	1.99	313.64	235.08	0.000
Psych8	Feeling depressed	2.10	1.54	309.00	243.00	0.000
Psych9	Periods of anxiety	1.87	1.49	301.12	260.40	0.001
Psych10	Panic attacks	1.33	1.13	294.38	263.73	0.000
Psych11	Feelings of hopelessness	1.99	1.51	307.93	240.32	0.000
Psych12	Feelings of life being meaningless	1.87	1.42	304.78	243.09	0.000
Psych13	Cannot stop worrying about being ill	2.10	1.80	292.98	265.18	0.039
Psych14	Low self-confidence	1.97	1.57	300.08	252.34	0.000
Psych15	Low self-esteem	1.91	1.58	296.90	260.03	0.004
Psych16	Able to laugh at things*	4.27	4.38	283.72	289.03	0.707
Psych17	Able to look forward to things*	3.93	4.27	271.09	308.05	0.009
Psych18	Difficulties talking about your illness to family/close friends	1.60	1.56	217.43	224.62	0.491
Psych19	Feeling that others think you talk too much about your illness	1.38	0.78	289.67	191.32	0.000
Psych20	Mental health affecting QoL	2.48	1.71	199.77	139.89	0.000
Psych21	Worrying about psychological/mental health	1.90	1.36	306.00	247.29	0.000

* Reversely coded response scale

**Table 11:** Means and mean rank sums for the domain on ADL—Comparison between ICU survivors and controls at the time of completing the questionnaire

	Issues	Mean		Mean rank sum		*p*-value
		ICU Survivors	Controls	ICU Survivors	Controls	
ADL1	Help getting dressed	1.43	1.06	303.30	266.28	0.000
ADL2	Help moving between chair and bed	1.27	1.03	299.95	273.01	0.000
ADL3	Support sitting up	1.14	1.02	294.51	280.97	0.007
ADL4	Help visiting the toilet	1.31	1.02	297.34	270.96	0.000
ADL5	Help with shopping	1.93	1.07	295.25	216.89	0.000
ADL6	Help with cooking	1.66	1.07	308.74	247.70	0.000
ADL7	Help with housework	2.05	1.09	321.09	223.33	0.000
ADL8	Help with medication	1.62	0.31	313.83	187.79	0.000
ADL9	Avoided travelling in a car	1.12	1.04	280.26	267.84	0.020
ADL10	Avoided taking public transport	1.41	1.08	224.63	200.09	0.000
ADL11	Help managing bills	1.63	1.06	305.80	253.90	0.000
ADL12	Daily activities affecting QoL	2.49	1.47	182.46	107.89	0.000
ADL13	Worrying about daily activities	2.08	1.29	320.40	228.34	0.000

**Table 12:** Means and mean rank sums for the domain on sleep—Comparison between ICU survivors and controls at the time of completing the questionnaire

	Issues	Mean		Mean rank sum		*p*-value
		ICU Survivors	Controls	ICU Survivors	Controls	
Sleep1	Need for daytime nap	2.21	1.62	313.56	238.38	0.000
Sleep2	Difficulties going to sleep	1.98	1.61	304.12	258.43	0.001
Sleep3	Need for sleeping pills	1.91	1.29	308.91	250.48	0.000
Sleep4	Anxiety before going to sleep	1.39	1.10	300.85	256.46	0.000
Sleep5	Difficulties going back to sleep	2.04	1.91	290.62	284.26	0.641
Sleep6	Night-time worrying	1.73	1.61	289.78	285.93	0.772
Sleep7	Nightmares	1.38	1.17	301.10	263.29	0.000
Sleep8	Nightly sweats disturbing sleep	1.41	1.33	291.80	278.94	0.251
Sleep9	Heart palpitations disturbing sleep	1.18	1.10	288.00	276.18	0.138
Sleep10	Sleep problems affecting QoL	2.16	1.66	235.99	184.82	0.000
Sleep11	Worrying about sleep	1.59	1.39	295.30	270.40	0.027

**Table 13:** Means and mean rank sums for the domain on appetite and alcohol use—Comparison between ICU survivors and controls at the time of completing the questionnaire

	Issues	Mean		Mean rank sum		*p*-value
		ICU Survivors	Controls	ICU Survivors	Controls	
A&A1	Bothersome thirst	1.78	1.26	309.00	252.29	0.000
A&A2	Difficulties chewing	1.43	1.08	302.81	264.77	0.000
A&A3	Sugar cravings	2.26	2.16	293.26	285.04	0.560
A&A4	Poor appetite	1.72	1.25	310.10	253.19	0.000
A&A5	Alcohol, how often	2.30	2.96	255.27	348.50	0.000
A&A6	Alcohol, how many glasses on a typical day	2.31	2.24	210.66	205.34	0.633
A&A7	Alcohol, how often 6 or more glasses	1.49	1.47	275.37	282.89	0.521
A&A8	Appetite affecting QoL	1.91	1.22	141.88	97.53	0.000
A&A9	Worrying about your appetite	1.34	1.09	299.26	264.46	0.000
A&A10	Alcohol affecting QoL	1.15	1.10	211.06	209.65	0.772
A&A11	Worrying about alcohol	1.14	1.14	282.87	292.09	0.203

**Table 14:** Means and mean rank sums for the domain on sexual health—Comparison between ICU survivors and controls at the time of completing the questionnaire

	Issues	Mean		Mean rank sum		*p*-value
		ICU Survivors	Controls	ICU Survivors	Controls	
Sex1	Difficulties handling physical closeness from loved ones	1.34	1.16	289.33	270.60	0.041
Sex2	Sex drive	3.47	2.82	299.92	221.88	0.000
Sex3	Sexual activity*	1.77	2.12	255.93	311.62	0.000
Sex4	Sex life	3.83	3.28	282.88	215.98	0.000
Sex5	Orgasm*	2.16	2.89	245.12	313.33	0.000
Sex6	Bothered by being naked in front of partner	1.39	1.24	210.11	198.59	0.152
Sex7	Surgical scars affecting sex life	1.66	1.14	172.73	138.35	0.000
Sex8	Lack of energy affecting sex life	2.06	1.76	268.63	241.12	0.025
Sex9	Pain during sex	1.23	1.10	151.17	139.14	0.026
Sex10	Problems with sex life affecting QoL	2.30	1.84	177.94	145.19	0.002
Sex11	Worrying about sex life	1.67	1.49	278.26	262.94	0.179

*Reversely coded response scale

**Table 15:** Means and mean rank sums for the domain on sensory functions—Comparison between ICU survivors and controls at the time of completing the questionnaire

	Issues	Mean		Mean rank sum		*p*-value
		ICU Survivors	Controls	ICU Survivors	Controls	
Sens1	Reduced taste	1.60	1.24	301.38	252.33	0.000
Sens2	Reduced smell	1.52	1.38	288.87	277.28	0.328
Sens3	Sound hypersensitivity	1.77	1.68	289.28	283.88	0.682
Sens4	Difficulties hearing what people say	1.94	2.06	278.28	298.40	0.144
Sens5	Reduced hearing limiting social life	1.36	1.40	282.41	287.18	0.656
Sens6	Sound hypersensitivity limiting social life	1.34	1.22	290.36	269.70	0.040
Sens7	Tinnitus	1.61	1.81	275.44	305.62	0.014
Sens8	Mouth dryness	1.93	1.35	311.96	235.31	0.000
Sens9	Mouth soreness	1.57	1.18	303.58	247.94	0.000
Sens10	Hoarseness	1.55	1.26	301.68	254.56	0.000
Sens11	Cracking voice	1.47	1.17	301.45	252.19	0.000
Sens12	Throat pain	1.33	1.14	294.37	264.87	0.002
Sens13	Throat feeling constricted	1.51	1.19	300.04	252.18	0.000
Sens14	Choking easily	1.42	1.19	296.30	259.59	0.000
Sens15	Difficulties swallowing	1.42	1.12	304.08	251.98	0.000
Sens16	Throat problems limiting social life	1.48	1.21	104.95	92.35	0.050
Sens17	Problems from sensory organs affecting QoL	2.10	1.62	225.93	171.82	0.000
Sens18	Worrying about your sensory organs	1.69	1.46	295.83	266.74	0.016

**Table 16:** Means and mean rank sums for the domain on gastrointestinal functions—Comparison between ICU survivors and controls at the time of completing the questionnaire

	Issues	Mean		Mean rank sum		*p*-value
		ICU Survivors	Controls	ICU Survivors	Controls	
GI1	Stoma	1.08	1.02	295.86	278.45	0.003
GI2	Constipation	1.80	1.63	295.69	273.01	0.087
GI3	Diarrhoea	1.93	1.57	300.99	262.69	0.004
GI4	Bowel urgency	1.82	1.55	295.38	267.92	0.034
GI5	Bowel leakage	1.36	1.15	296.76	266.67	0.003
GI6	Bowel problems limiting social life	1.44	1.11	187.36	162.20	0.001
GI7	Bowel problems affecting QoL	2.12	1.57	188.55	146.54	0.000
GI8	Worrying about bowel problems	1.60	1.27	297.06	265.61	0.005

**Table 17:** Means and mean rank sums for the domain on urinary functions—Comparison between ICU survivors and controls at the time of completing the questionnaire

	Issues	Mean		Mean rank sum		*p*-value
		ICU Survivors	Controls	ICU Survivors	Controls	
UT1	Difficulties feeling the need to urinate	1.28	1.06	294.47	269.54	0.001
UT2	Difficulties emptying the bladder	1.49	1.31	291.03	272.00	0.092
UT3	Night-time emptying of bladder	2.39	2.27	293.22	272.08	0.126
UT4	Urinary urgency	1.63	1.58	288.40	278.47	0.437
UT5	Stress incontinence	1.61	1.53	208.72	208.10	0.954
UT6	Urinary problems limiting social activities	1.34	1.05	203.27	180.32	0.000
UT7	Urinary problems affecting QoL	1.72	1.47	189.83	170.21	0.049
UT8	Worrying about urinary problems	1.48	1.40	286.82	287.35	0.962

**Table 18:** Means and mean rank sums for the domain on work life—Comparison between ICU survivors and controls at the time of completing the questionnaire

	Issues	Mean		Mean rank sum		*p*-value
		ICU Survivors	Controls	ICU Survivors	Controls	
Work1	Same type of work in 2 years' time	2.00	1.69	148.21	101.97	0.000
Work2	Capacity for work and physical demands	3.94	3.21	268.14	154.45	0.000
Work3	Capacity for work and psychological demands	3.57	2.88	259.49	162.66	0.000
Work9	Work problems affecting QoL	3.00	2.63	107.11	76.60	0.000
Work10	Financial problems affecting QoL	3.67	2.74	116.06	69.81	0.000
Work11	Worry about future working life	2.38	1.94	240.81	209.20	0.003
Work12	Worry about future work capacity	2.42	2.08	247.13	200.36	0.000
Work13	Worry about future finances	3.15	2.24	271.78	213.25	0.000

*Cognitive difficulties* affecting quality of life such as losing the thread easily, think you have done something you haven’t, mistaken which day of the week it is, or difficulties taking initiatives.

*Fatigue* was a common symptom often with the need for a daytime rest. It could be tough getting started doing things, difficult finishing things due to feeling exhausted or doing things under pressure of time or multitasking. Getting tired from reading, or from conversation between more than two people could affect work and limit social activities.

*Physical health* in general was more often affected among ICU survivors. They could suffer from reduced body feeling, muscle weakness in arms or legs, dizziness when standing up, losing balance easily, difficulties climbing stairs, unsteady gait, contractures and shortness of breath, many of them limiting physical activities.

*Pain* was reported from different parts of the body or as general body pain by survivors making painkillers necessary for managing ADL or to get sufficient sleep.

*Psychological health* problems were also overrepresented among the survivors. It made them cry more easily. Feeling low-spirited or depressed, or suffering from panic attacks, were also more common as was feelings of hopelessness and feelings of life being meaningless. Many suffered from low self-confidence.

*Activities of daily living (ADL)* were often more difficult for the survivors. They could need help with things like getting dressed, moving from bed to chair, visiting the toilet, shopping, cooking and doing housework. Help with medication and managing bills was also more common.

*Sleep* could be affected in many ways, at worst as nightmares.

*Poor appetite*, bothersome thirst and difficulties chewing more often affected quality of life for the ICU-survivors. Together with reduced taste, mouth dryness, mouth soreness or mouth pain, swallowing were difficult and made it easier to choke.

*Sexual health issues* like libido and sex life were less satisfactory in ICU-survivors.

*Work life* differed between the two groups. For the survivors the capacity for work was negatively affected both due to physical demands and psychological demands. Work problems as well as financial problems were more common among ICU-survivors.

A complete list of all questions and their response rates is shown in Additional file 2: table S2a/Additional file 3: S2b. All continuous variables were found to deviate from normality in both groups. No additional valuable information was added to the space after each domain.

## Discussion

Our study describes a first step toward an intensive care long-term follow-up questionnaire with the capacity to detect the burden of ICU survivorship and the effect on quality of life. By creating a questionnaire from interviews with ICU survivors and testing it on ICU survivors and a non-ICU-treated control group, we were able to show that the questionnaire contained most issues experienced by survivors and was able to identify differences between the two groups. While issues found in our interviews may apply to a general population to a certain degree, it is our belief that many might worsen after intensive care. A comparison with a non-ICU-treated control group may help describe to what extent intensive care can be attributed to a change in magnitude rather than simply describe a prevalence.

At a stakeholders' conference in 2010, the concept of PICS was created to enclose impairments in mental health, cognition and physical functions [6]. At a follow-up conference in 2012, the PICS group pointed out the need for outcome assessment tools created with qualitative methods [25]. Several groups have addressed this issue, either by developing new instruments or by examining the evidence of content validity of existing ones. Jeong and Kang reported the development and validation of a questionnaire specifically for the three domains of PICS, using a methodology similar to ours [26]. In 2018, Nedergaard et al*.* interviewed 18 ICU survivors and extracted the most important issues [27]. Although symptoms from the PICS domains were well represented, additional symptoms were also considered important, for example incontinence, short temper and the feeling of being isolated. Furthermore, large differences between patients and clinicians when ranking the importance of symptoms have been found in areas as diverse as bariatric surgery [28], diabetes [29] and aphasia [30]. These findings would argue toward instruments developed with input from former patients.

Regarding measuring HRQoL (Health-Related Quality of Life), SF-36 and EQ-5D are currently the most commonly used tools after intensive care. Lim et al. extracted post-ICU issues from 30 ICU survivors and let the same patients compare these issues with SF-36 and EQ-5D [8]. Of the domains identified as relevant by the ICU survivors, only one was considered adequately covered by SF-36 or EQ-5D. The remaining domains were either inadequately covered or completely missing, suggesting that the use of either of these instruments as a measurement of post-ICU HRQoL will miss important issues. In another study, Jensen et al. were unable to show improvement in HRQoL measured by SF-36 after their ICU recovery program and recommend new instruments to be developed and validated to assess the particular HRQoL problems of post-ICU patients [31].

Our questionnaire focuses on different measures of ICU survivorship: First of all, it covers the areas of PICS; physical, cognitive and mental health. Secondly, it describes quality-of-life related to the survivor's health status. Further, areas not covered by PICS or SF-36/EQ-5D such as dysphagia [32], joint contractions [33], sleep disturbances [34] and personal finances [35] are included, all previously described problems after intensive care.

### Strengths

The response rate of 76.2% from the ICU survivors and 85.3% from the control group indicates not only the usability of the questionnaire in a trial context, but that questions were considered relevant. Participants did not provide any additional issues in the comment areas in the questionnaire, only encouraging comments, arguing toward evidence of content validity in our questionnaire rather than "questionnaire fatigue."

The development of the questionnaire follows international recommendations for development of patient-reported outcome measures [36]. Choosing interviewees purposively instead of in a consecutive order has been the most effective for reaching data saturation with minimum sample size in simulations [37] Data saturation is the most commonly used delimiter for sample size but not randomizing the order of the interviews poses a hypothetical risk of affecting the saturation point [38]. Therefore, we decided à priori to set the sample size to when three consecutive interviews did not provide any new information.

We took several steps to show evidence of content validity: First, we based this provisional questionnaire mainly on issues reported by ICU survivors themselves. Second, all interviewees were read the field notes to ensure our proper understanding of issues. Third, we used cognitive interviews, and finally we allowed all participants to add potentially missing issues in the quantitative phase.

### Limitations

This being a provisional questionnaire awaiting further analyses, there are some limitations. Even if a high response rate indicates the relevance of the questions asked, this questionnaire will be reduced. Differences in comorbidities between ICU survivors and the control group, where 13 of 22 differed significantly, align with previous findings that chronic comorbidities are common in ICU patients [39]. We do not know to what extent these comorbidities explain the differences between the two groups. The questionnaire has not been developed from, nor tested on, patients with a shorter time from ICU discharge than six months; hence, our questionnaire may miss issues that resolve completely within this time frame. Nor was the questionnaire developed for patients with an ICU length-of-stay shorter than 72 h or with neurological/neurosurgical primary diagnoses, and results cannot be generalized to these groups. We cannot exclude that interviewees may have forgotten issues experienced between ICU discharge and the interview, and thus our questionnaire cannot claim to be comprehensive. However, by including all issues appearing in interviews, no matter how uncommon, we have attempted to minimize the impact of potential recall bias. In the second phase, recall bias was accounted for by using ‘the last month’ as time frame in the provisional questionnaire. Although we have shown that a majority of issues differed significantly in magnitude in comparison with a non-ICU-treated control group, we do not know to what extent these differences were already prevalent before intensive care. Regarding internal validity, there was a difference in age between the interviewees and the cohort groups. However, ranges of age, SAPS score etc. did not differ markedly. Finally, we cannot exclude a selection bias with regard to patients who chose not to participate or who we were unable to reach.

## Conclusions

This study describes the development of a provisional questionnaire for long-term health-related quality of life and burden of disease after intensive care. This first version, based mainly on issues from interviews with ICU survivors, clearly identified burden of disease affecting multiple domains in a large group of patients. The next steps in order to make this questionnaire a useful tool for follow-up after intensive care include further statistical analyses including psychometric properties and reduction in the number of questions.

## Supplementary Information

**Additional file 1.** **Table S1**: List of scales and questionnaires discussed during interviews with ICU survivors.

**Additional file 2.** **Table S2a**: Descriptive statistics and response rates for ICU survivors and the control group.

**Additional file 3.** **Table S2b**: Descriptive statistics and response rates for ICU survivor-specific questions.

**Additional file 4.** **Figure S1**: Examples of interview questions and verbal prompts and probes used in both original interviews and during cognitive interviews.

**Additional file 5.** **Figure S2**: Examples on construction of questions.

## Data Availability

The datasets generated during and/or analyzed during the current study are not publicly available due to them containing information that could compromise research participants privacy/consent, but are available from the corresponding author on reasonable request.

## Abbreviations

ADL: Activities of daily living
EQ-5D: EuroQoL's five dimensions instrument
ICU: Intensive care unit
HRQoL: Health-related quality of life
PICS: Post-intensive care syndrome
PTSD: Post-traumatic stress disorder
SAPS 3: Simplified acute physiology score
SF-36: Short-form (36) health survey
