# Supplementary material for: Long-term health-related quality of life and burden of disease after intensive care: development of a patient-reported outcome measure
Source: Crit Care. 2021 Feb 25;25:82. doi: 10.1186/s13054-021-03496-7 (PMC7905420; doi:10.1186/s13054-021-03496-7)
Supplement: Supplementary file 1 — Additional file 1. Table S1: List of scales and questionnaires discussed during interviews with ICU survivors. [file 13054_2021_3496_MOESM1_ESM.docx]

Table S1 - List of scales and questionnaires discussed during interviews with ICU survivors.

AUDIT-C (Alcohol Use Disorders Identification Test-Consumption)

ESAS (Edmonton Symptom Assessment Scale)

EQ-5D (EuroQol 5 dimensions)

FSFI (Female Sexual Function Index)

FSMC (Fatigue Scale for Motor and Cognitive Functions)

HADS (Hospital Anxiety and Depression Scale)

HHIA (Hear Handicap Inventory for Adults)

FAS (Fatigue Assessment Scale)

FACIT (Functional Assessment of Chronic Illness Therapy)

FSS (Fatigue Severity Scale)

IES-R (Impact of Event Scale revised)

Katz ADL (Katz Index of Independence in Activities of Daily Living)

MSAS (Memorial Symptom Assessment Scale)

Pfeffer FAQ (Pfeffer Functional Activities Questionnaire)

PSQI (Pittsburg Sleep Quality Index)

PCL-5 (PTSD Checklist)

Sexual Dysfunction Questionnaire

SF-36 (Short Form 36)

WAI (Work Ability Index)
