# Supplementary material for: Long-term health-related quality of life and burden of disease after intensive care: development of a patient-reported outcome measure
Source: Crit Care. 2021 Feb 25;25:82. doi: 10.1186/s13054-021-03496-7 (PMC7905420; doi:10.1186/s13054-021-03496-7)
Supplement: Supplementary file 4 — Additional file 4. Figure S1: Examples of interview questions and verbal prompts and probes used in both original interviews and during cognitive interviews. [file 13054_2021_3496_MOESM4_ESM.docx]

**Figure S1 – Examples of interview questions and verbal prompts and probes used in both original interviews and during cognitive interviews.**

1. We are asking for your help in creating a questionnaire which will be used to identify and follow the experiences of patients who have survived intensive care. I would like to ask you a few things about your health. Can you tell me about the experiences you may have had as a result of your intensive care stay, and the time between discharge and today?
2. Would you mind telling me about a typical day when you are/have X?
3. Have you learned to manage X?
4. Would you mind telling me about your thoughts or feelings when you think about X?
5. Is there anything else you think I should know of to understand X in more detail?
6. If you can, would you mind trying to tell me how you define it, so I have it in your words?
7. How do you feel when X?
8. In what way?; Just how do you mean?; Could you give me an example?; Would you tell me a little more about that?
9. [*Once the interviewee can think of nothing else*] In addition to what we have talked about, I would like to tell you about some areas that others before you have talked about, to see if it evokes any thoughts in you.
10. [*Additional question during cognitive interviews*] Can you think of anything else that you have experienced or had to cope with after your intensive care stay that is not included in this questionnaire?

**Prompts used to focus on certain areas:**

1. In what ways have you changed since the ICU discharge?
2. What are you not able to do that you were able to do before your intensive care stay, and why?
3. Are you limited in normal daily activities or self-care compared to before your intensive care stay? What is it that limits you?
4. Has X affected your social life/work life in any way?
5. Has X affected your quality of life in any way?
6. Who has provided the most support for you since your ICU discharge? In what way has he or she been helpful?
7. Have any changes in your relationships with family/friends occurred?
8. Do you have financial problems or worries due to your intensive care stay?
9. Have your personal feelings/emotional wellbeing changed?
