# Supplementary material for: Long-term health-related quality of life and burden of disease after intensive care: development of a patient-reported outcome measure
Source: Crit Care. 2021 Feb 25;25:82. doi: 10.1186/s13054-021-03496-7 (PMC7905420; doi:10.1186/s13054-021-03496-7)
Supplement: Supplementary file 5 — Additional file 5. Figure S2: Examples on construction of questions. [file 13054_2021_3496_MOESM5_ESM.docx]

**Figure S2 - Examples on construction of questions.**

| **Quotes** | **Domains and issues** | **Questions and response scales** |
| --- | --- | --- |
| *”…and you know, I keep putting up Post-It notes, like everywhere…”*  Male, 57 years old | ***Cognitive function*** Coping with memory problems | Have you used notes or other tools in your daily living to help you to not forget, the last month?   - No - Rarely - Sometimes - Quite often - Very often - All the time |
| *“…you know once I even fell asleep while talking to her.”*  Male, 51 years old | ***Fatigue*** Difficulties staying awake during activities | Have you falling asleep while talking to someone, the last month?   - No - Rarely - Sometimes - Quite often - Very often - All the time |
| *“…they haven’t been able to explain it, my fingers get all white and red from temperature, and it hurts…”*  Female, 68 years old | ***Physical health***  Physical manifestations of reduced peripheral circulation | Have your fingers whitened from cold, or turned red and swollen from temperature shifts, the last month?   - No - Rarely - Sometimes - Quite often - Very often - All the time |
| *“It’s so strange, sometimes even normal touch can feel like needles!”*  Female, 56 years old | ***Pain*** Hypersensitivity | Have you found normal touch bothersome, the last month?   - Not at all - A little - Moderately - Quite a bit - Very much |
| *“…you know it’s absolutely horrible, I can’t even, like, feel warmth towards them, and it hurts me so much…”*  Female, 59 years old | ***Psychological aspects***  Difficulties bringing forward known emotions | Have you had difficulties feeling warmth towards family members, the last month?   - No - Rarely - Sometimes - Quite often - Very often - All the time |
| *“…and you know, in the beginning, since my bus passes the exact spot where we crashed, I got panic attacks, so now you know, I make my dad drive me everywhere so we can go a different way…”*  Male, 38 years old | ***Activities of daily living (ADL)*** Restrained in social activities | Have you refrained from public transporting because of feelings of uneasiness, the last month?   - No - Occasionally - Half of the times - Most of the times - Every time |
| *“These nightmares…I don’t know how to describe them, they’re more than nightmares, so much more real, more like memories…”*  Female, 82 years old | ***Sleep***  Nightmares | Have you had nightmares, difficult to separate from reality, the last month?   - No - Occasionally - Half of the nights - Most of the nights - Every night |
| *“[Partner] You know, nowadays you keep a chocolate bar close to you like everywhere you go. [Interviewee] I know, it’s just you know, the urge, the craving, it just hits me.”*  Male, 70 years old | ***Appetite and alcohol*** Sweet craving | Have you experienced sweet cravings, the last month?   - No - Rarely - Sometimes - Quite often - Very often - All the time |
| *“…but can you imagine, I don’t even want to be naked in front of him. All the scars, I hate my body, it’s not mine, it’s not me!”*  Female, 42 years old | ***Sexual health*** Self-image and other intimacy issues | Have you been bothered by being naked in front of partner, the last month?   - Not at all - A little - Moderately - Quite a bit - Very much |
| *“...and it’s so weird, the voice, it, like, it crackles, it breaks, I don’t know how to describe it…”*  Female, 71 years old | ***Sensory functions*** Changes in voice | Have you experienced your voice crackling, without having a cold, the last month?   - Not at all - A little - Moderately - Quite a bit - Very much |
| *“…all these pills, I feel, it seems my stomach, you know, I can’t you know, I can’t go to the bathroom, it’s just, nothing happens…”*  Female, 20 years old | ***Gastrointestinal functions*** Bowel functions and dysfunctions | Have you experienced constipation, the last month?   - No - Occasionally - Once a week - Several times a week - Once a day - Several times a day |
| *“I never had these problems before, but once I got home from the hospital, it was like I can’t feel when I have to go, you know what I mean?”*  Male, 64 years old | ***Urinary functions*** Urinary dysfunctions | Have you experienced difficulties feeling the need to urinate, the last month?   - No - Occasionally - half of the times - most of the times - Every time |
| *“We couldn’t really afford me staying home anymore. I didn’t feel ready, but we just couldn’t afford it.”*  Male, 57 years old | ***Work life*** Effect on personal finances | Were you forced to return early to work for financial reasons? [*Question only for the ICU survivor group*]   - No - Yes |
